# Hotspots and future trends of dermatofibrosarcoma protuberans

**DOI:** 10.3389/fonc.2024.1399486

**Published:** 2024-11-12

**Authors:** Zhen Meng, Rui Zhang, Zhihong Sun, Cong Fu, Zhiyu Li, Luying Wang, Ran Huo, Feng Xue

**Affiliations:** ^1^ Department of Plastic and Aesthetic Surgery, Shandong Provincial Hospital, Shandong University, Jinan, Shandong, China; ^2^ Department of Plastic and Aesthetic Surgery, Shandong Provincial Hospital Affiliated to Shandong First Medical University, Jinan, Shandong, China

**Keywords:** dermatofibrosarcoma protuberans, fibrosarcomatous transformation, bibliometric analysis, fusion gene, tumor microenvironment, immunotherapy

## Abstract

**Objective:**

Dermatofibrosarcoma protuberans (DFSP) is a moderately malignant soft tissue sarcoma with localized infiltrative growth. The extensive surgical scope and high recurrence rate of DFSP brings dysfunctional, aesthetic, psychological and economic problems to patients. The aim of this study is to explore the global publication characteristics, research hotspots and future trends of DFSP over the past 32 years via an intuitive visualized way.

**Methods:**

The Web of Science Core Collection (WoSCC) database was searched for papers related to DFSP published from 1990 to 2022. Then bibliometric analysis of these publications was performed, including collaborative networks, co-citation analysis of journals and references, and cluster analysis of keywords.

**Results:**

A total of 1588 papers were retrieved between 1990-2022. The United States was the most prolific country, followed by China. The article Imatinib Mesylate in Advanced Dermatofibrosarcoma Protuberans: Pooled Analysis of Two Phase II Clinical Trials, received most citations. Research hotspots and future trends are mainly focused on disease diagnosis, COL1A1-PDGFB fusion gene, drug and surgical treatment, fibrosarcomatous transformation, and immunotherapy.

**Conclusion:**

The research on DFSP faces several clinical challenges. This study provides novel insights into future research directions and scientific decisions for DFSP.

## 1 Introduction

Dermatofibrosarcoma protuberans (DFSP), also known as Darier Ferrand tumor (62), is a rare malignant tumor of the skin and subcutaneous tissue with a incidence of approximately 4.2–5.0 per million people per year in the United States (1, 2, 61). DFSP is the most common type of cutaneous sarcomas (63, 64) with more than 6,000 new cases diagnosed annually (3). According to the 2020 World Health Organization classification of soft tissue sarcomas, DFSP is recognized as moderately malignant (locally invasive) sarcomas (4). The tumor grows slowly with localized finger-like infiltrative growth in the dermis or subcutaneous tissue. It’s rarely metastasizes but easily recurs (65, 66). The risk factors for DFSP are relatively unknown, but about 10%– 20% of DFSP cases were associated with prior trauma of any type. Older patients were likely to develop DFSP at the trauma site, which was more frequently located on the face and lower legs (5). Several histological subtypes of DFSP often coexist with a high degree of tissue heterogeneity, making it difficult for early and accurate diagnosis (67). Secondary delayed treatment or overtreatment can be traumatic and economically burdensome for patients. Although the tumor-related mortality rate is low, its infiltrative growth pattern leads to extensive surgical scope, dysfunction and aesthetic problems after surgical resection. In addition, the high recurrence rate often accompanies with multiple surgeries, which causes psychologicaland financial burdensome. Moreover, poor prognosis has led to an increasing emphasis on the clinical management of DFSP.

Bibliometric analysis provides a comprehensive analysis of publications and reveals the research status in a particular field. It is characterized by the ability to visualize the contributions of different countries, authors, and journals in a particular field. Unlike traditional reviews, it performs a powerful function in predicting research prospects. In recent years, bibliometric analysis has been widely used in the field of medicine, particularly in the field of oncology. However, there have been no detailed bibliometric studies of DFSP. This study aims to retrospectively analyze the literature of past 32 years from 1990 to 2022, derive the current research status and research hotspots, and provide new perspectives for future research directions and scientific decisions.

## 2 Materials and methods

### 2.1 Data source and searching strategy

The Web of Science database is a high-quality database that covers publications in different fields and has complete literature-related and citation data, making it the most suitable database for bibliometric studies (68, 69). In this paper, Web of Science Core Collection (WoSCC) database (SCI-expanded Index) was selected as the data source. The literature related to DFSP (1990-01-01 to 2022-12-31) was searched using the following search strategy: TS=(“dermatofibrosarcoma* protuberan*”) OR TS=(“dermatofibrosarcoma*”) OR TS=(“DFSP”) OR TS=(“darier ferrand tumor”). We restricted the article type to article or review and the language to English. To avoid database updating bias, all literature searching and data extraction were done on 2023.08.21. Plain text files including fully documented and cited references were downloaded from the database.

### 2.2 Screening strategy

Two researchers screened the literature by title, abstract, keywords and full text independently. After the initial screening two researchers cross-checked, and a third researcher was involved in resolving disagreements when necessary, and finally reached a consensus on the exclusion of some of the literature. Exclusion criteria: 1. non-peer-reviewed articles: e.g. conference papers, letters, comments 2. irrelevant to DFSP: e.g. delayed FS predictability (DFSP) (6) or Function and Service Discovery Protocol (DFSP) (7) 3. veterinary themes (except disease models). Diagnosis, differential diagnosis, and therapeutic approaches such as: mohs surgery, flaps, and some type of soft tissue sarcomas were not excluded because they are closely related to DFSP.

### 2.3 Data analysis

VOSviewer 1.6.19 and CiteSpace 6.2.R3 were used to perform visualization analysis of authors and co-cited authors, co-citation analysis of journals, and clustering analysis of keywords. CiteSpace6.2.R3 was used to perform visualization analysis of countries/regions and institutions, co-citation analysis of references and citation burst analysis, as well as draw the dual-map overlay of journals. The Bibliometrix R was used to analyze core journals and trend topics.

### 2.4 Ethics and consent

This study does not involve animal or human subjects and thus does not require ethical approval.

## 3 Results

### 3.1 General information

**Figure 1:**
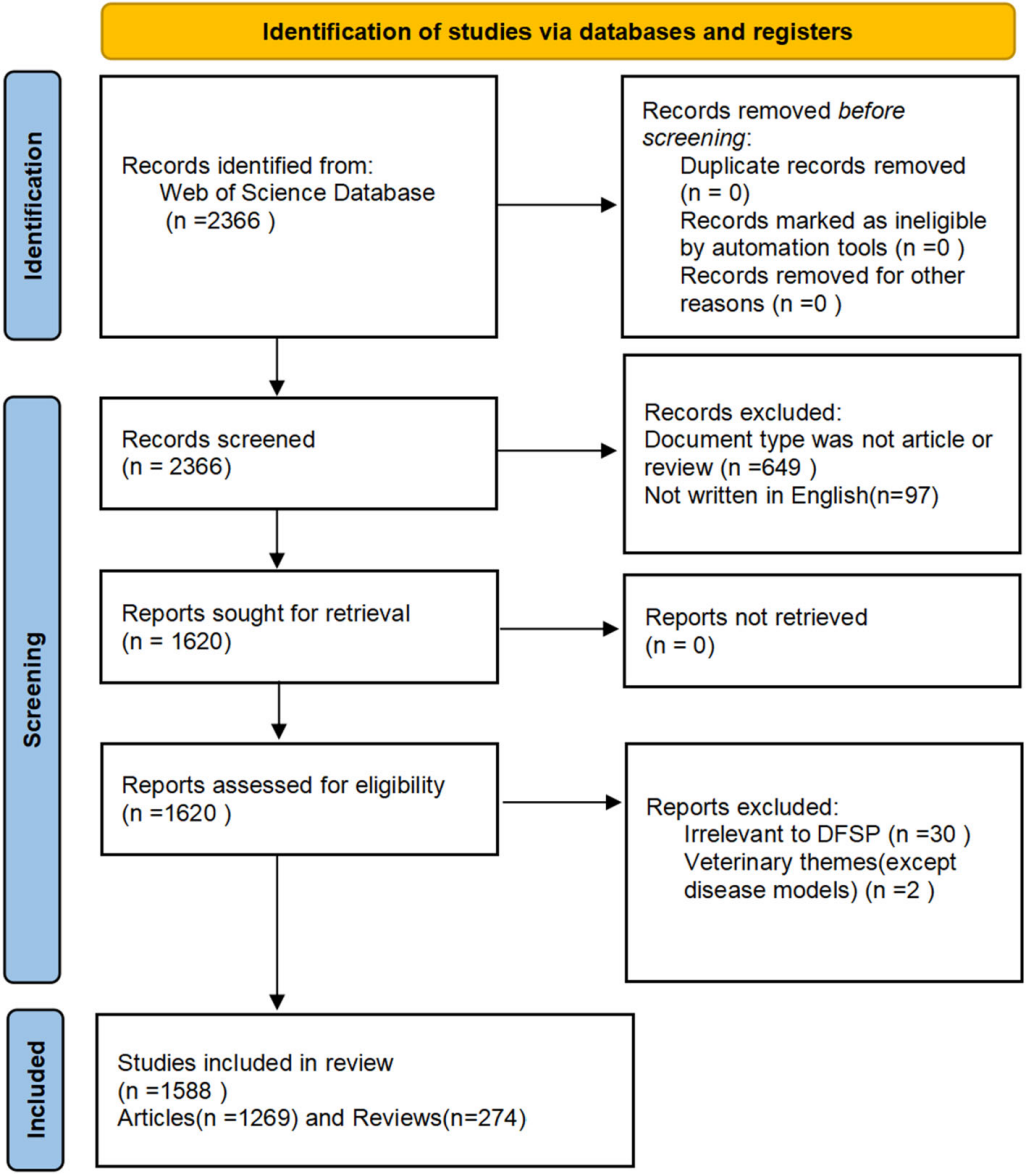
The PRISMA flow diagram.

According to the PRISMA flow diagram of the present study(**Figure 1**), a total of 1,588 papers published between 1990 and 2022 were included.

### 3.2 Annual publication outputs

**Figure 2:**
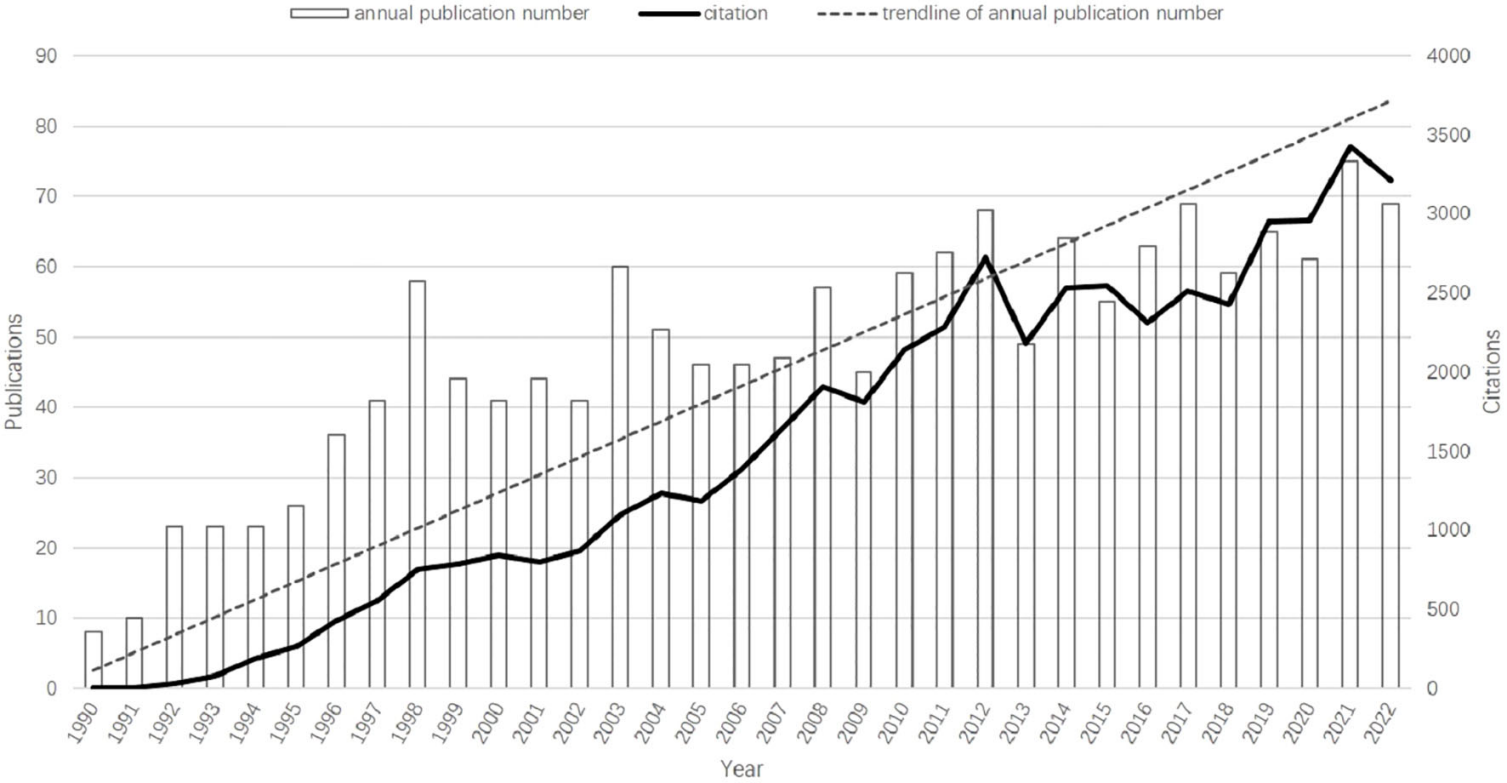
Trends in the growth of publications and the number of citations.

The overall growth trend of the annual number of publications and citation frequency was shown in **Figure 2**. There is a rapid growth in the number of publications from 8 to 58 in 1990–1998, a stable period in 1998–2013, and even a negative growth in a few years, and a steady growth in 2013–2022. The publications increase from 8 (in 1990) to 69 (in 2022), and at the same time the number of citations increase from 2 times (in 1990) to 3210 (in 2022). 75 papers published in 2021,which peaked the annual number of publications.

### 3.3 Contribution of countries/regions and institutions

**Figure 3:**
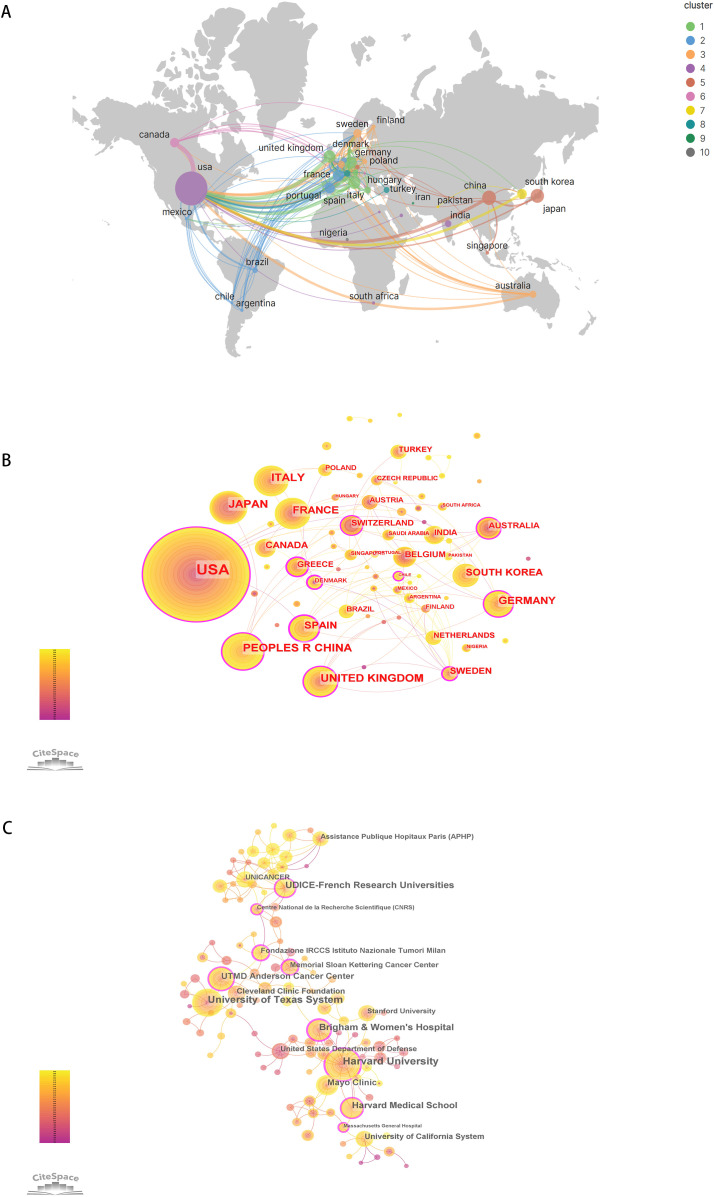
The collaboration of countries/regions and institutions in the field of DFSP. **(A)** The distribution and collaboration map of countries/regions regarding the research of DFSP. **(B)** Co-occurrence network of countries/regions. **(C)** Co-occurrence network of institutions.

During these 32 years, 73 countries/regions and 1746 institutions have been conducted research on DFSP. As shown in **Table 1**, the United States was the most prolific country, followed by China and Japan. These three countries together contributed more than half of the total number of publications. Italy, United Kingdom, Germany, and France also contributed relatively more to the field. The national geographic visualization map drawn by VOSviewer and Scimago Graphica showed the cooperation relationship and the amount of publications between countries/regions (**Figure 3A**). There were 36 countries/regions with no less than 4 publications. Except Nigeria and Iran, all the countries/regions have closer cooperation with each other, especially in Europe and the U.S.A. The circles in CiteSpace represent the countries/regions, and the lines represent their cooperation (**Figure 3B**). Purple circles refer to countries with high betweeness centrality (greater than or equal to 0.1), indicating that these countries were important in the research network. It showed that USA, China, UK and Germany were the countries with both a high number of publications and high betweenesss centrality, while Japan and Italy only had a high number of publications. Denmark and Chile, on the contrary, do not have a high number of publications but have established collaborations with many countries.

**Table 1:** Top 10 productive countries/regions and Institutions regarding the research of DFSP.

Rank	Countries/regions	Count(%)	Centrility	H-index	Rank	Institution(country)	Count(%)	Centrility	H-index
**1**	USA	688(43.325%)	0.20	89	1	HARVARD UNIVERSITY (USA)	86(5.416%)	0.24	34
**2**	PEOPLES R CHINA	120(7.556%)	0.13	21	2	UNIVERSITY OF TEXAS SYSTEM (USA)	77(4.849%)	0.05	28
**3**	JAPAN	116(7.305%)	0.00	27	3	UDICE FRENCH RESEARCH UNIVERSITIES (FRANCE)	59(3.715%)	0.20	26
**4**	ITALY	102(6.423)	0.04	32	4	BRIGHAM WOMEN S HOSPITAL (USA)	53(3.338%)	0.28	29
**5**	United Kingdom	100(6.297%)	0.12	36	5	HARVARD MEDICAL SCHOOL (USA)	53(3.338%)	0.11	27
**6**	GERMANY	90(5.668%)	0.11	29	6	UTMD ANDERSON CANCER CENTER (USA)	48(3.023%)	0.11	23
**7**	FRANCE	82(5.164%)	0.04	32	7	UNICANCER (FRANCE)	44(2.771%)	0.03	23
**8**	SPAIN	68(4.282%)	0.28	22	8	MAYO CLINIC (USA)	41(2.582%)	0.06	22
**9**	SOUTH KOREA	58(3.652)	0.08	16	9	MEMORIAL SLOAN KETTERING CANCER CENTER (USA)	36(2.267%)	0.32	23
**10**	CANADA	54(3.401%)	0.04	25	10	UNIVERSITY OF CALIFORNIA SYSTEM (USA)	36(2.267%)	0.09	20

Harvard University ranked first with 86 publications. University of Texas System (77), UDICE-French Research Universities (59), Brigham & Women’s Hospital (53), Harvard Medical School (53) also contributed significantly to DFSP research (**Table 1**). Eight of the top ten ranked institutions belong to the United States. In terms of collaboration between institutions (as shown in **Figure 3C**), we found that the institution with a high degree of centrality (circled in purple) including Memorial Sloan Kettering Cancer Center, Brigham & Women’s Hospital, Harvard University, the Fondazione IRCCS Istituto Nazionale Tumori Milan, and UDICE-French Research Universities were concentrated in Europe and the United States.

### 3.4 Analysis of authors and co-cited authors

There were 6312 authors involved in the research field of DFSP over the past 32 years, and **Table 2** listed the top ten most productive authors. The most productive author was Fletcher,Christopher D.M. (Department of Pathology, Brigham and Women’s Hospital, USA), followed by Mentzel,Thomas(Department of Dermatopathology Friedrichshafen,Germany), and Pedeutour, Florence (Laboratory of Solid Tumors Genetics and Institute for Research on Cancer and Aging of Nice (IRCAN), France).

**Table 2:** TOP10 productive and co-cited authors in the field of DFSP.

Rank	Author	Documents	Citations	Average citation/publication	Country	Rank	Co-Cited author	Country	H-index	Co-citations
**1**	Fletcher, Christopher D. M.	31	2897	93.45	USA	1	Fletcher, Christopher D. M.	USA	25	392
**2**	Mentzel, Thomas	24	1163	48.46	GERMANY	2	Mentzel, Thomas	GERMANY	12	391
**3**	Pedeutour,Florence	23	1533	69.68	FRANCE	3	Gloster, HM	USA	2	350
**4**	Goldblum, John R.	16	1075	67.19	USA	4	Enzinger, FM	USA	2	288
**5**	Bridge, Julia A.	13	496	38.15	USA	5	Pedeutour, Florence	FRANCE	10	288
**6**	Coindre,Jean-Michel	13	1078	82.92	FRANCE	6	Weiss, SW	USA	4	271
**7**	Hornick, Jason L.	13	1280	98.46	USA	7	Simon, Marie-Pierre	FRANCE	8	267
**8**	Sanmartin, Onofre	13	453	34.85	SPAIN	8	Goldblum, John R.	USA	13	249
**9**	Fisher, Cyril	12	940	78.33	ENGLAND	9	Taylor, SC	USA	1	223
**10**	Serra-Guillen,Carlos	12	449	37.42	SPAIN	10	Bowne, WB	USA	1	209

Authors with more than 5 publications were defined as core authors according to the Price law. The collaboration network of these 99 core authors was classified into 21 clusters based on the closeness of their connections (**Supplementary Figure 1A**). When combined with the superimposed visualization of Year (**Supplementary Figure 1B**), it reflected that green, purple and pink, were predominantly active before 2000, and that orange, light blue and red clusters have published the most articles in the last 10 years. The top ten co-cited authors were also listed in **Table 2**. Fletcher, Christopher D.M. was the most co-cited author(392), followed by mentzel Thomas (391), and Gloster, HM (Department of Dermatology, University of Cincinnati, USA)(350). Co-citation network was shown in the cluster plot(**Supplementary Figure 1C**), and the impact of co-cited author was shown in the density plot (**Supplementary Figure 1D**).

### 3.5 Journals and co-cited journals

A total of 439 journals reported scholarly results on DFSP and the top ten most productive journals were shown in **Table 3**. Journal of Cutaneous Pathology (98, 6.171%) was the most productive journal, followed by American Journal of Dermatopathology (61, 3.841%) and Journal of the American Academy of Dermatology (52, 3.275%). Among the top ten most productive journals, eight were from the United States, one from Denmark, and one from the United Kingdom. Four had an impact factors>5, including Journal of the American Academy of Dermatology (13.8), Modern Pathology (7.5), Histopathology (6.4), and American Journal of Surgical Pathology (5.6).

**Table 3:** Top 10 productive and co-cited journals in the field of DFSP.

Rank	Journal (productive)	Publications	Citations	Average citation/publication	Country	Impact Factor(2022)	JCR	Rank	Journal(co-cited)	Co-citations	Country	Impact Factor(2022)	JCR
**1**	Journal of Cutaneous Pathology	98	2341	23.89	Denmark	1.7	Q3	1	American Journal of Surgical Pathology	3979	USA	5.6	Q1
**2**	American Journal of Dermatopathology	61	1282	21.02	USA	1.1	Q4	2	Cancer	3003	USA	6.2	Q1
**3**	Journal of the American Academy of Dermatology	52	2943	56.60	USA	13.8	Q1	3	Journal of the American Academy of Dermatology	2690	USA	13.8	Q1
**4**	American Journal of Surgical Pathology	45	4559	101.31	USA	5.6	Q1	4	Journal of Clinical Oncology	1803	USA	45.3	Q1
**5**	Dermatologic Surgery	45	1252	27.82	USA	2.4	Q2	5	Journal of Cutaneous Pathology	1574	Denmark	1.7	Q3
**6**	International Journal of Dermatology	30	412	13.73	USA	3.6	Q1	6	Dermatologic Surgery	1343	USA	2.4	Q2
**7**	Modern Pathology	29	2658	91.66	USA	7.5	Q1	7	American Journal of Dermatopathology	1322	USA	1.1	Q4
**8**	Pediatric Dermatology	27	305	11.30	USA	1.5	Q4	8	Histopathology	1301	England	6.4	Q1
**9**	Cancer Genetics and Cytogenetics	24	832	34.67	USA	1.929	Q3	9	Cancer Research	1202	USA	11.2	Q1
**10**	Histopathology	23	901	39.17	England	6.4	Q1	10	Modern Pathology	1038	USA	7.5	Q1

It was reported that the impact of a journal in a field depends on the number of papers published and the number of citations they receive (8). The co-citation analysis of journals was performed by VOSviewer and it could help us to understand the most influential journals in a particular focus area. The co-cited journals were categorized into four clusters (**Supplementary Figure 2A**), with the red representing mainly dermatology journals, such as Journal of the American Academy of Dermatology, Dermatologic Surgery. And the green represented academic journals in the field of oncology, such as Journal of Clinical Oncology, Cancer Research. Blue for pathology journals such as American Journal of Surgical Pathology, Journal of Cutaneous Pathology, Histopathology. Yellow for cytogenetic journals, such as Genes Chromosome & Caner, Cancer Genetics and Cytogenetics. American Journal of Surgical Pathology (IF5.6, Q1) was the official journal of the Arthur Purdy Stout Society of Surgical Pathologists and The Gastrointestinal Pathology Society. It was the most influential journal with 3979 co-citations, demonstrating a high level of authority in the field of surgery and pathology. Among the top ten most co-cited journals, seven had an impact factors>5, of which the Journal of Clinical Oncology (IF=45.3, Q1) was the most highly cited journal with the highest impact factor, demonstrating the high quality of articles related to DFSP and the academic significance of this study (**Table 3**).

A dual-map overlay of journals was used to represent the relationship between citing and cited journals, with citing journals on the left side and cited journals on the right side, and different colored paths indicating different citation relationships. As shown in **Supplementary Figure 2B**, four main paths were identified. These shifting trajectories showed that the disciplinary center of the journals moved from health, nursing, medicine, molecular biology, and genetics to molecular, biology, immunology, medicine, medical, clinical and dentistry, dermatology, surgery. Journal source dynamics was shown in **Supplementary Figures 2C, D**, which showed the annual and cumulative appearances of the ten journals with the highest number of publications in the field. According to Bradford’s Law, a total of 16 core journals were identified (**Supplementary Figure 2E**), including Journal of Cutaneous Pathology, American Journal of Dermatopathology, Journal of the American Academy of Dermatology, American Journal of Surgical Pathology, Dermatologic Surgery, etc.

### 3.6 Co-cited references

Co-cited references were analyzed using Citespace. The article “Imatinib Mesylate in Advanced Dermatofibrosarcoma Protuberans: Pooled Analysis of Two Phase II Clinical Trials” ranked first with a co-citation frequency of 56. Diagnosis and treatment of DFSP were the most popular topics in these highly cited papers, particularly targeted therapy with imatinib and choice of surgical method. We performed a co-citation network and cluster analysis using CiteSpace (**Supplementary Figures 3A, B**). References were categorized into 19 clusters, such as recurrence(#0), wide local excision(#1), molecular targeted therapy(#3), CD34(#5), FISH(#6), micrographic surgery(#7), translocation(#10), COL1A1-PDGFB fusion transcripts(#11), PDGFRA(#12), sti-571(#13), tissue microarray(#17). The top 25 references with the strongest citation bursts was shown in **Supplementary Figure 3C**. Among these references, eight have experienced citation bursts in recent years and may predict future trends in DFSP research. Five were on diagnosis and treatment; one addressed prognosis; one discussed FS-DFSP, and one explored pathology and cytogenetics.

### 3.7 Bibliometric analysis of keywords

**Figure 4:**
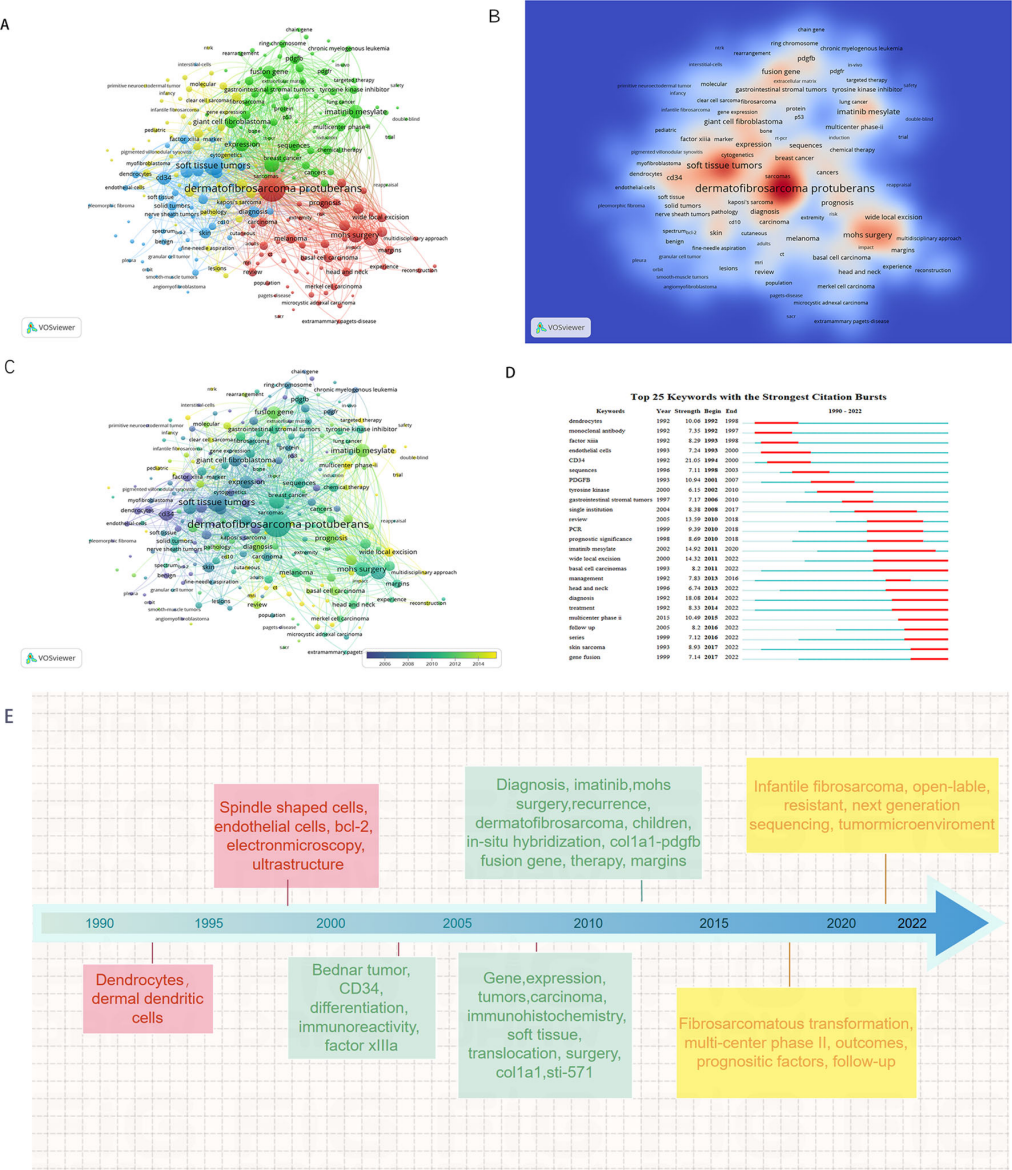
Keyword analysis in the field of DFSP. **(A)** VOSviewer cluster visualization of keywords. **(B)** VOSviewer density visualization of keywords. **(C)** VOSviewer overlay visualization of keywords. **(D)** Top 25 keywords with the strongest citation bursts. **(E)** VOSviewer overlay visualization of keywords display on timeline.

Keyword co-occurrence network was constructed and visualized by VOSviewer with All Keywords (**Figure 4A**). As shown in **Figure 4A**, the keywords could be divided into four clusters: 1) The pathogenesis of DFSP was associated with fusion genes owing to chromosomal translocation rearrangement, which promoted tumor growth by activating the platelet-derived growth factor (PDGF) receptor. Imatinib inhibited tumor growth via this receptor. (Green cluster including “fusion gene,” “chromosomal translocations,” “imatinib mesylate,” “pdgf,” “ring chromosome,” “col1a1-pdgfb fusion transcripts”); 2) The most important treatment for DFSP was surgery, and the choice of surgical approach and postoperative follow-ups were important factors for tumor prognosis and management. (Red cluster including “DFSP,” “surgery,” “mohs surgery,” “flap,” “reconstruction,” “recurrence,” “wide local excision,” “managemen,t” “margins,” “prognosis”); 3) DFSP was a moderately malignant STS with multiple histological subtypes and pathological similarities to other soft tissue tumors, requiring differential diagnosis. (blue cluster including “soft tissue tumors,” “diagnosis,” “differential diagnosis,” “cd34,” “ immunohistochemistry,” “factor xiiia,” “ histopathology”); 4) Cytogenetics was a definitive diagnostic method when it’s difficult to diagnose by clinical presentation and histopathology. (Yellow cluster including “cytogenetics,” “pcr,” “fluorescence *in situ* hybridization,” “molecular”). Keywords density was visualized in **Figure 4B**. The higher the keywords weight, the closer the color is to red. We also visualized the average year of occurrence using different colors, as shown in **Figure 4C**. Purple indicates earlier keywords and yellow indicates recent keywords. In addition, the top 25 keywords with the strongest citation bursts was constructed by Citespace and shown in **Figure 4D**, which indicated a rapid increase in research topic during that period, suggesting that this might be a popular topic and trend for that time. The emergent words during 1992–2000, 1998–2010, and 2010–2022 were CD34 (21.05), PDGFB (10.94), and diagnosis (18.08), respectively. **Figure 4E** displays the keywords evolution timeline.

**Figure 5:**
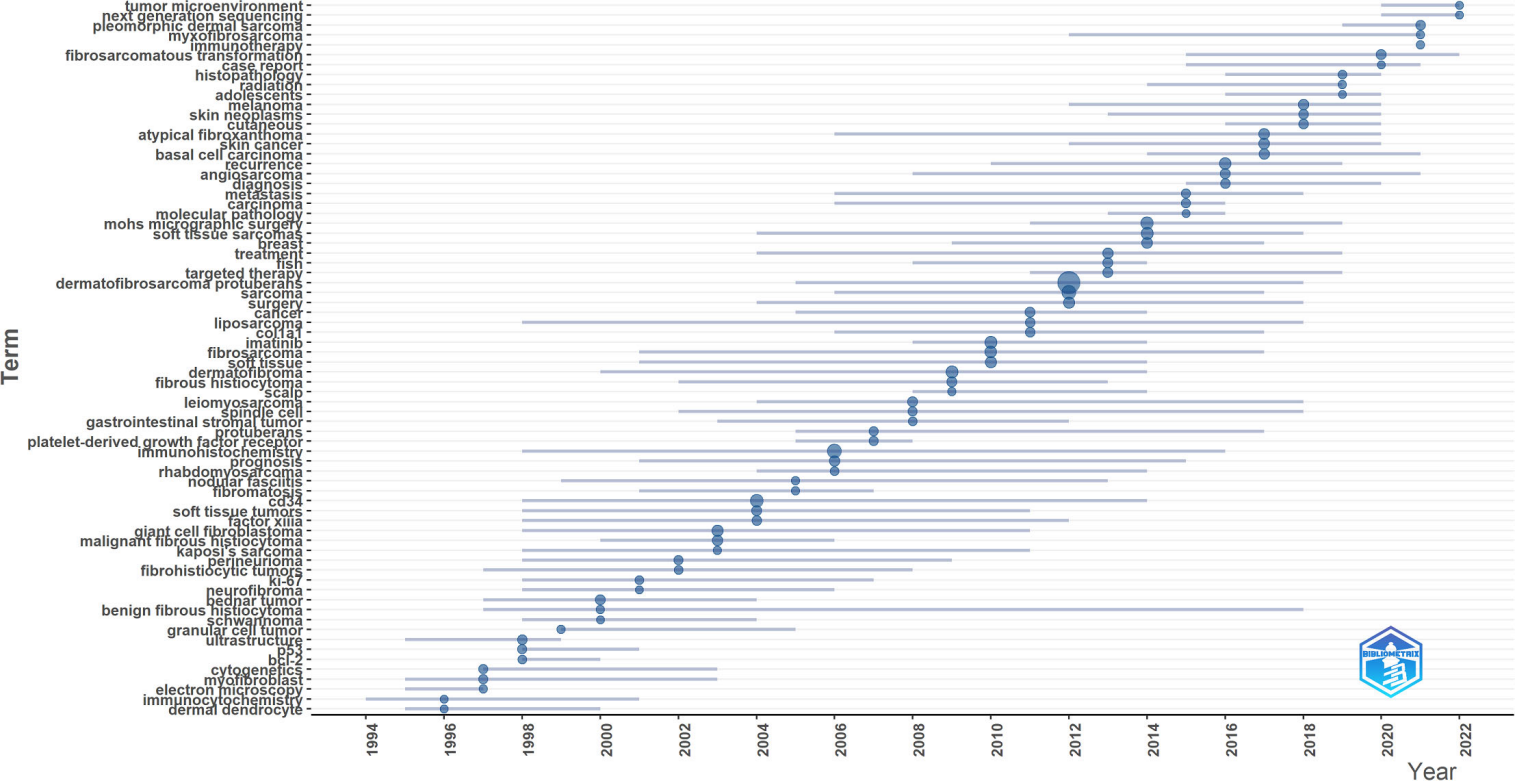
Topic dynamics in the field of DFSP.

**Figure 5** showed the dynamics of the theme per year. During 1990–2009, the terms “immunohistochemistry,” “CD34,” “Factor IIIa,” “ki-67,” “bcl-2” suggested that the research theme in this phase was the diagnosis and differential diagnosis of DFSP. This involved the use of immunohistochemistry methods with markers such as CD34, Bcl-2, Factor IIIa, and ki-67%. While during 2010–2014, the terms “FISH,” “COL1A1,” “imatinib” suggested that the research hotspot in this phase was the detection of the COL1A1-PDGFB fusion gene by FISH. Additionally, they confirmed the effectiveness of imatinib. In the period of 2015–2020, the terms “fibrosarcomatous transformation,” “histopathology,” “recurrence,” “metastasis” suggested that tumor recurrence and metastasis and relationship with FS-DFSP were the research focus in this period. During 2021–2022, the terms “tumor microenvironment,” “next generation sequencing,” “immunotherapy” suggested that the recent popular topics was finding new target molecules and promoting target therapy through next-generation sequencing (NGS) technology, as well as to promote the development of immunotherapy through the study of tumor microenvironment.

## 4 Discussion

### 4.1 General information

In this study, a total of 1588 papers related to DFSP were identified and included for bibliometric analysis. Results found that 6312 authors from 1746 institutions were involved in research field of DFSP.

The annual publication of DFSP-related research has steadily increased over the past 30 years, indicating that an increasing number of scholars are focusing on this topic. Before 1990, there had been little research on DFSP. The discovery of CD34, ring chromosome (9–11), chromosomal translocation t(17;22)(q22;q13) (12, 13) and the COL1A1-PDGFB fusion gene (14) in 1990s led to a rapid growth of publications on DFSP from 1990–1998. In 2002, WHO published a new classification of STS (15) and updated it in 2013 and 2020 (4, 16, 17). In 2006, imatinib was approved by the Food and Drug Administration (FDA) for the treatment of unresectable, recurrent, and metastatic DFSP (18). In the past decade, there has been a rapid increase in the number of DFSP-related publications. As the diagnostic rate has been increased, the targeted drug imatinib has become clinically available, and sequencing technology has been updated.

China, the United States, and European countries have numerous publications and rank highly in terms of their research value and influence. Asian and African countries have significantly less cooperation than European and American countries. Owing to the advantages of developed countries in disease management and resource sharing, the establishment of national oncology centers and the systematization and standardization of oncology treatment processes have brought developmental advantages to scientific research. As for institutions, Sichuan University, Fudan University, Shanghai Jiao Tong University and Sun Yat-sen University have gradually established international academic cooperation. European scholars (Spain and Italy) have been more active in collaborating in the field of DFSP in the last 5 years. For example, Sanmartin, Onofre (Spain) has contributed to the study of Mohs surgery (19–24), while Gronchi, Alessandro (Italy) has contributed to immunotherapy research (25–28).

American Journal of Surgical Pathology was the most influential journal, based on the combined results of publication output and citations (8). In addition, some oncology and dermatology journals, such as the Journal of the American Academy of Dermatology, Journal of Clinical Oncology, Cancer, and Cancer Research, provided a large number of high-quality research suggesting the DFSP’s multidisciplinary management model.

### 4.2 Research hotspots and future trends

According to Price law, the reference pattern marks the frontiers of scientific research (29). Keywords are the author’s summary of the paper, and keywords analysis can effectively summarize the hotspots and frontiers of the field. Given the co-citation analysis of references, combined with citation burst analysis and keyword clustering analysis. The hotspots and frontiers of the DFSP are summarized as follows.

### 4.3 Rapid and accurate diagnosis of DFSPs

The diagnosis and differential diagnosis of DFSP are important to avoid undertreatment and overtreatment. DFSP was classified into fibroblastic/myofibroblastic tumors based on histopathological type (4). In 1992, Setsuya et al. at the Tohoku University first discovered that the expression of the CD34 could be used as a new method to distinguish DFSP from other fibrohistiocytic tumors (30). CD34 expression is positive or strongly positive in DFSP (31, 32). In addition, DFSP can also show weak aberrant expression of EMA and can be positive for H3K27me3 and GRIA2, but negative for bcl-2, S-100 protein, desmin, STAT6, KIT(CD117), and ERG (33–36). However, both the microscopic and immunohistochemical features mentioned above are not specific. For example, plaque-like CD34-positive dermal fibroma, sclerotic fibroma and solitary fibrous tumors may show similar cellular arrangement features and positive immunohistochemistry for CD34 (37–39). In a previous study, 9.1% of DFSP patients were CD34 negative, especially FS-DFSP (40). With improvements in cytogenetic technology, RT-PCR for chimeric gene transcripts and FISH for PDGFB gene rearrangement are effective for cases that are difficult to diagnose (41). The COL1A1-PDGFB fusion gene is detected in over 90% DFSP cases, making it highly specific for diagnosis (42). The annual topic dynamics suggest that NGS enabling the identification of new markers for clinical diagnosis and treatment. In 2022, a team from the Ninth People’s Hospital of Shanghai Jiaotong University identified a novel biomarker, TKL2, which can be used for diagnosis and treatment through RNA sequencing (43).

### 4.4 Fusion genes and derivative ring chromosomes

Genetically, 90–96% or more of DFSPs characteristically show chromosomal translocation rearrangement t (17; 22)(q22; q13) and ring chromosomes that result in COL1A1-PDGFB gene fusion (14, 70). This encodes a fusion protein that functionally overlaps with the mature form of PDGFB and promotes tumor growth by activating the PDGFB receptor through autocrine-paracrine effects (71–73). Gene fusion occurring on two different forms of chromosomes is a special feature of DFSP. Therefore, the detection of fusion genes can provide useful diagnostic and prognostic information. However, there are approximately 4%–10% of DFSP cases in which the COL1A1-PDGFB fusion gene is not detected. Among these, approximately 40% may have a cryptic COL1A1-PDGFB gene fusion, and the other 55% may be associated with PDGFD. Brendan C. Dickson et al. detected a new gene fusion—the COL6A3-PDGFD gene fusion—and found an apparent predilection for breast (74).

The trend topics suggest that next-generation sequencing (NGS), a cost-effective technology widely used in various diseases, enabled identification of new markers for clinical diagnosis and treatment of DFSP. Nolan Maloney et al. identified a novel gene fusion, MAP3K7CL-ERG, by RNA sequencing in one case of FS-DFSP, which may be associated with the transformation of DFSP to FS-DFSP (75). In 2022, Cong Peng et al. performed the first whole-genome sequencing of DFSP at Xiangya Hospital in China and identified a novel gene fusion, SLC2A5-BTBD7, in DFSP [t(1;14)] (76). The identification of novel fusion genes and translocations presents a new potential target for diagnostic and therapeutic interventions in DFSP. The molecular complexity of DFSP is evident, and future studies on its genome are required to discover new genomic aberrations as potential disease diagnostic and therapeutic targets.

### 4.5 Imatinib targeted therapy

Surgery is the primary treatment for DFSP, but non-surgical methods are necessary for unresectable, recurrent, or metastatic cases. DFSP is less effective against general chemotherapy; therefore, targeted therapy is a priority (77). Imatinib mesylate (STI571), is a selective kinase inhibitor that inhibits the activation of ALB, KIT, PDGFRA, and PDGFRB, thereby inhibiting tumor growth (78, 79). Imatinib was initially used to treat chronic granulocytic leukemia and gastrointestinal mesenchymal stromal tumors (GIST). In 2002, Brian P. Rubin et al. first reported the efficacy of imatinib in treating patients with metastatic and inoperable DFSP (80). Imatinib is clinically active against both localized and metastatic DFSP with chromosomal translocations; however, cases without fusion genes may not respond to imatinib (44, 45). This suggests that genetic detection is required before targeted treatment to predict the clinical response to imatinib.Although most cases of localized DFSP can be effectively managed through surgical intervention, imatinib may play a supportive role in disease control for patient spresenting with locally advanced or metastatic conditions, including those with DFSP exhibiting fibrosarcomatous components (46).

Imatinib failed to provide adequate tumor regression in some patients and exhibited secondary resistance. Several studies have suggested that low PDGFB phosphorylation may be associated with imatinib resistance (73, 81, 82). Moderate to strong activation of EGFR and insulin receptors has been detected in some patients with DFSP, suggesting that therapeutic drugs other than PDGFRB may target these complex kinase receptors (82). Pazopanib exerts antiangiogenic effects by inhibiting of VEGFR and acting on the PDGFB receptor. Japanese researchers found that Akt-mTOR pathway-related proteins were activated in DFSP and that the Akt-mTOR pathway is a potential therapeutic target in imatinib-resistant DFSP or FS-DFSP (81). Grant Eilers et al. discovered that CDKN2A deletion can contribute to DFSP progression. CDK4/6 inhibition is a preclinically effective treatment against p16-negative, imatinib-resistant FS-DFSP, and should be evaluated as a therapeutic strategy in patients with unresectable or metastatic imatinib-resistant DFSP (83). Future research directions include exploring the mechanism of imatinib resistance and identifying other targeted therapeutic agents.

### 4.6 Choice of surgical procedure

The finger-like invasive growth pattern of DFSP makes it difficult to determine the tumor boundary, leading to a high recurrence risk (47). As previously stated,Surgical intervention is the primary treatment for DFSP, encompassing marginal excision, wide local excision (WLE) and Mohs micrographic surgery (MMS). Marginal resection is not recommended because of its high recurrence rate (84). Unlike conventional vertical sections, MMS allows the microscopic examination of continuous horizontal sections, enabling clean resection with maximum preservation of normal tissue, which may require only a 1 cm surgical margin (85). Regardless of the surgical approach, resection of the DFSP should be as complete as possible (R0 resection) to reduce the rate of local recurrence.

Some studies have shown that the MMS results in lower recurrence rates (86, 87). Currently, local recurrence after tumor resection ranges from 26% to 60%. WLE can reduce this to 0%-41%, while MMS can control it to 0%-8.3% (48). The 2023 NCCN guidelines discuss the recurrence rate of MMS and WLE, which is approximately 0%–6.6% of MMS compared to 1.7%–30.8% of WLE (49, 66, 88–93). A meta-analysis of 684 DFSP patients found recurrence rates of 9.10% after WLE and 2.72% after MMS (50). At the Mayo Clinic, Lowe et al. reported a 30.8% recurrence rate after WLE and 3.0% after MMS (49). However, the high dose of local anesthesia and the time-consuming handling of frozen sections limit the widespread use of MMS (66). Some pathologists believe that MMS is inaccurate because the tiny DFSP tissues remaining in paraffin sections cannot be distinguished from the scattered spindle cells in the normal dermis, scar, and connective tissues, and the results of CD34 staining are highly variable in frozen sections (94–96). Traditional MMS is ineffective for observing DFSP tumor cells because the larger tumor size and frozen tissue can extend operation time and increase stroke risk. Additionally, tumor cells often contain excess fat, leading to incomplete freezing and potential false negatives. Therefore, we prefer modified slow MMS (3, 51). For areas like the face and neck,especially in children where extensive resection is unsuitable, MMS or slow MMS is recommended for better reconstruction and aesthetics (52, 53). When Mohs surgery is not possible, a wide local excision with a guaranteed depth of resection combined with the help of a pathologist can also achieve complete resection (97). If postoperative routine pathology suggests a positive margin (R1 resection), most scholars believe that secondary resection should be performed immediately, while some scholars have suggested that patients with DFSP should be followed up closely. However, for patients with FS-DFSP, immediate secondary resection is recommended to achieve negative margins. The choice of the surgical approach remains controversial. The extent and surgical approach will remain the focus of future research.

In recent years, intraoperative navigation techniques, such as near-infrared fluorescence (NIRF) imaging, have been used to identify tumor margins and assist in complete tumor resection (98). This aligns with modern precision medicine, enabling accurate assessment of tumor margins and detection of residual lesions during surgery.The study of intraoperative navigation systems is a future research direction.

### 4.7 Identification and significance of fibrosarcomatous transformation

Fibrosarcomatous transformation represents the transition from classic DFSP to spindle cell fascicle proliferation, with approximately 10%–20% of DFSP transforming into FS-DFSP and the proportion is progressively higher with increasing recognition (54, 63, 99–102). Numerous studies have shown that FS-DFSP has a higher rate of recurrence, metastasis, and death. The presence of fibrosarcomatous areas is an independent poor prognostic factor (55, 63, 103, 104). MARCOVAL et al. reported that fibrosarcomatous areas may raise recurrence risk to 14-52% (54, 56, 57). Generally, Fewer than 5% of patients with DFSP develop distant metastases (58, 59). It is higher in FS-DFSP(FS-DFSP14.4% vs DFSP 1.1%) (55). Advanced age, female sex, and large tumor size are risk factors for FS-DFSP and are relevant for early identification (102). Patients with FS-DFSP should be managed according to the guidelines for soft-tissue sarcomas. Multimodal treatment and postoperative surveillance, including lymph nodes in the drainage area and chest computed tomography, are proposed (61).

The underlying genetic mechanisms of fibrosarcomatous transformation remain poorly understood. A study by Japanese researchers suggested that alterations in the PDGFR-Akt-mTOR pathway may be associated with the progression of DFSP to FS-DFSP (81). In 2018, Bérengère Dadone-Montaudié et al. detected the presence of the fusion gene EMILIN2-PDGFD in two cases without the COL1A1-PDGFB fusion gene, along with a CDKN2A homozygous deletion, all of which were present in the fibrosarcomatous area, which may indicate an increased malignant potential of DFSP (105). In 60 report a DFSP with fibrosarcomtous morphology harboring a novel TNC-PDGFD fusion (60). In 2022, Yang Lu et al. from West China Hospital identified co-amplification of 12q15 and 12p13, as well as CDKN2A/2B deletion, in one case of FS-DFSP; these genetic aberrations were confined to the fibrosarcomatous component, suggesting a synergistic role in the progression to sarcoma (106). Mechanistic studies and the early identification of FS-DFSP are future research trends.

### 4.8 Tumor microenvironment and immunotherapy

Tumor microenvironment (TME) is the surrounding microenvironment in which tumor cells exist. It comprises cellular components and an extracellular matrix (ECM). Interactions between tumor cells and the tumor microenvironment are crucial for tumor cell growth, invasion, and metastasis. In addition, immune cells in the microenvironment can play tumor-suppressive or tumor-promoting roles. It’s reported that high concentrations of matrix metalloproteinase (MMP) in fibrosarcoma tissues led to high degradation of the extracellular matrix, which promoted tumor growth and metastasis, and controlling MMP activity could regulate tumor growth and metastasis. *In vitro* experiments on fibrosarcoma confirmed that intrathecal injection of the MMP inhibitor TIMP-1-GPI inhibited cell proliferation and migration, increased apoptosis, and enhanced sensitivity to chemotherapeutic agents (107). In a study of imatinib for metastatic DFSP, Italian researchers found that imatinib treatment had an effect on the tumor microenvironment, including increased endothelial cell permeability and increased immune infiltration of NK cells and B cells (28). A study conducted at the Ninth People’s Hospital of Shanghai Jiaotong University analyzed the immune microenvironment of DFSP. The results indicated that the infiltration of Th2 cells and macrophages increased in tumor tissues, whereas that of CD8+ T cells, Th1 cells, and NK cells was not significantly different from that in normal tissues. Cancer-associated fibroblasts were significantly upregulated in DFSP and are expected to be an intervention target for inhibiting DFSP invasion (43).

### 4.9 Limitation

First, the literature used for this study was obtained from the WoSCC database, we did not search for additional databases, and many studies were omitted because they were published in non-SCI journals or other databases. Also, the databases are updated in real time, and this study may differ from the actual amount of literature. Second, CiteSpace and VOSviewer cannot completely replace systematic searches. Third, bibliometrics cannot assess the quality of individual studies, and the variable quality of the literature may reduce the credibility of the analysis. Because citation metrics are time-dependent, earlier articles tend to be cited more often than recent articles. Despite these limitations, they have less impact on the major trends presented in this paper. Overall, our study provides a basis for understanding the current status, hotspots, and future trends in the study of DFSP.

## 5 Conclusions

Abundant studies on DFSP have been conducted over the past 32 years, and the number of annual publication has steadily increased. The United States was the most prolific country, followed by China. Research hotspots and future research trends are primarily in disease diagnosis, COL1A1-PDGFB fusion gene, drug and surgical treatment, fibrosarcomatous transformation, and immunotherapy. This study provides novel insights into future research directions in the field of DFSP.

## Data Availability

The raw data supporting the conclusions of this article will be made available by the authors, without undue reservation.

## References

[1] Allen A Ahn C Sangueza OP . Dermatofibrosarcoma Protuberans. Dermatologic Clinics. (2019) 37:483. doi: 10.1016/j.det.2019.05.006 31466588

[2] Hao XP Billings SD Wu FB Stultz TW Procop GW Mirkin G . Dermatofibrosarcoma Protuberans: Update on the Diagnosis and Treatment. J Clin Med. (2020) 9:22. doi: 10.3390/jcm9061752 PMC7355835 32516921

[3] Chen J Sun D Rao Y-M Zheng H-Y Gong X Xu H . Practice for multidisciplinary diagnosis and treatment of dermatofibrosarcoma protuberans: expert consensus of Shanghai Ninth People's Hospital, Shanghai Jiao Tong University School of Medicine, (2020 edition). J Of Shanghai Jiao Tong Univ (Medical Science). (2021) 41:1669–75. doi: 10.3969/j.issn.1674-8115.2021.12.018

[4] Sbaraglia M Bellan E Dei Tos AP . The 2020 WHO Classification of Soft Tissue Tumours: news and perspectives. Pathologica. (2021) 113:70–84. doi: 10.32074/1591-951X-213 33179614 PMC8167394

[5] Choi ME Lee M Lee WJ Won CH Chang SE Lee MW . Clinical and histopathological analysis of 141 dermatofibrosarcoma protuberans in Korea: A comparative study according to trauma. Australas J Dermatol. (2022) 63:E297–304. doi: 10.1111/ajd.13920 36066015

[6] Weinberger MJ Ordentlich E . On delayed prediction of individual sequences. IEEE Trans Inf Theory. (2002) 48:1959–76. doi: 10.1109/TIT.2002.1013136

[7] Ramírez PLG Taha M Lloret J Tomás J . An Intelligent Algorithm for Resource Sharing and Self-Management of Wireless-IoT-Gateway. IEEE Access. (2020) 8:3159–70. doi: 10.1109/Access.6287639

[8] Dzikowski P . A bibliometric analysis of born global firms. J Business Res. (2018) 85:281–94. doi: 10.1016/j.jbusres.2017.12.054

[9] Bridge JA Neff JR Sandberg AA . Cytogenetic Analysis of dermatofibrosarcoma protuberans. Cancer Genet Cytogenetics. (1990) 49:199–202. doi: 10.1016/0165-4608(90)90142-W 2208055

[10] Iwasaki H Ohjimi Y Ishiguro M Isayama T Fujita C Kaneko Y . Supernumerary ring chromosomes and nuclear blebs in some low-grade malignant soft tissue tumours: atypical lipomatous tumours and dermatofibrosarcoma protuberans. Virchows Archiv-an Int J Pathol. (1998) 432:521–8. doi: 10.1007/s004280050200 9672193

[11] Pedeutour F Coindre JM Sozzi G Nicolo G Leroux A Toma S . Supernumerary ring chromosomes containing chromosome-17 sequences - a specific feature of dermatofibrosarcoma protuberans. Cancer Genet Cytogenetics. (1994) 76:1–9. doi: 10.1016/0165-4608(94)90060-4 8076341

[12] Minoletti F Miozzo M Pedeutour F Sard L Pilotti S Azzarelli A . Involvement of chromosomes 17 and 22 in dermatofibrosarcoma protuberans. Genes Chromosomes Cancer. (1995) 13:62–5. doi: 10.1002/gcc.2870130110 7541645

[13] Pedeutour F Simon MP Minoletti F Barcelo G TerrierLacombe MJ Combemale P . Translocation, t(17;22)(q22;q13), in dermatofibrosarcoma protuberans: A new tumor-associated chromosome rearrangement. Cytogenetics Cell Genet. (1996) 72:171–4. doi: 10.1159/000134178 8978765

[14] Simon MP Pedeutour F Sirvent N Grosgeorge J Minoletti F Coindre JM . Deregulation of the platelet-derived growth factor B-chain gene via fusion with collagen gene COL1A1 in dermatofibrosarcoma protuberans and giant-cell fibroblastoma. Nat Genet. (1997) 15:95–8. doi: 10.1038/ng0197-95 8988177

[15] Toro JR Travis LB Wu HJ Zhu K Fletcher CDM Devesa SS . Incidence patterns of soft tissue sarcomas, regardless of primary site, in the Surveillance, Epidemiology and End Results program 1978-2001: an analysis of 26,758 cases. Int J Cancer. (2006) 119:2922–30. doi: 10.1002/ijc.v119:12 17013893

[16] Fletcher CDM Bridge JA Hogendoorn PCW Mertens F World Health O International Agency for Research on, C . WHO classification of tumours of soft tissue and bone. 4th edition. Lyon: IARC Press (2013).

[17] Lauer S Gardner JM . Soft tissue sarcomas-New approaches to diagnosis and classification. Curr Problems Cancer. (2013) 37:45–61. doi: 10.1016/j.currproblcancer.2013.03.001 23719330

[18] Mertens F Johansson B Fioretos T Mitelman F . The emerging complexity of gene fusions in cancer. Nat Rev Cancer. (2015) 15:371–81. doi: 10.1038/nrc3947 25998716

[19] Diago A Llombart B Serra-Guillen C Arana E Guillen C Requena C . Usefulness of ultrasound in dermatofibrosarcoma protuberans and correlation with histopathological findings: A series of 30 cases. Skin Res Technol. (2021) 27:701–8. doi: 10.1111/srt.13003 33455037

[20] Llombart B Serra-Guillen C Rubio L Nagore E Requena C Traves V . Subcutaneous dermatofibrosarcoma protuberans, a rare subtype with predilection for the head: A retrospective series of 18 cases. J Am Acad Dermatol. (2017) 77:503–511.e1. doi: 10.1016/j.jaad.2017.02.046 28420485

[21] Nieto-Benito LM Ciudad-Blanco C Sanmartin-Jimenez O Garces JR Rodriguez-Prieto MA Vilarrasa E . Mohs micrographic surgery in dermatofibrosarcoma protuberans: Rate and risk factors for recurrence in a prospective cohort study from the Spanish Registry of Mohs Surgery (REGESMOHS) and review of the literature. Exp Dermatol. (2021) 30:717–22. doi: 10.1111/exd.14291 33523531

[22] Rios-Vinuela E Serra-Guillen C Llombart B Requena C Nagore E Traves V . Pleomorphic dermal sarcoma: a retrospective study of 16 cases in a dermato-oncology centre and a review of the literature. Eur J Dermatol. (2020) 30:545–53. doi: 10.1684/ejd.2020.3875 33021478

[23] Rodriguez-Jimenez P Jimenez YD Reolid A Sanmartin-Jimenez O Garces JR Rodriguez-Prieto MA . State of the art of Mohs surgery for rare cutaneous tumors in the Spanish Registry of Mohs Surgery (REGESMOHS). Int J Dermatol. (2020) 59:321–5. doi: 10.1111/ijd.14732 31777957

[24] Serra-Guillen C Llombart B Nagore E Guillen C Sanmartin O . Determination of Margins for Tumor Clearance in Dermatofibrosarcoma Protuberans: A Single-Center Study of 222 Cases Treated With Modified Mohs Surgery. Dermatologic Surg. (2022) 48:51–6. doi: 10.1097/DSS.0000000000003269 34743125

[25] Baldi GG Gronchi A Tazzari M Stacchiotti S . Immunotherapy in soft tissue sarcoma: current evidence and future perspectives in a variegated family of different tumor. Expert Rev Anticancer Ther. (2022) 22:491–503. doi: 10.1080/14737140.2022.2065986 35412415

[26] Blay JY Hindi N Bollard J Aguiar S Angel M Araya B . SELNET clinical practice guidelines for soft tissue sarcoma and GIST. Cancer Treat Rev. (2022) 102:10. doi: 10.1016/j.ctrv.2021.102312 34798363

[27] Frezza AM Stacchiotti S Gronchi A . Systemic treatment in advanced soft tissue sarcoma: what is standard, what is new. BMC Med. (2017) 15:12. doi: 10.1186/s12916-017-0872-y 28571564 PMC5455204

[28] Tazzari M Indio V Vergani B De Cecco L Rini F Negri T . Adaptive Immunity in Fibrosarcomatous Dermatofibrosarcoma Protuberans and Response to Imatinib Treatment. J Invest Dermatol. (2017) 137:484–93. doi: 10.1016/j.jid.2016.06.634 27608549

[29] Price DJ . Networks of Scientific Papers. Science. (1965) 149:510–5. doi: 10.1126/science.149.3683.510 14325149

[30] Aiba S Tabata N Ishii H Ootani H Tagami H . Dermatofibrosarcoma protuberans is a unique fibrohistiocytic tumor expressing-CD34. Br J Dermatol. (1992) 127:79–84. doi: 10.1111/j.1365-2133.1992.tb08036.x 1382538

[31] Abenoza P Lillemoe T . CD34 and Factor-Xiiia in the differential-diagnosis of dermatofibroma and dermatofibrosarcoma protuberans. Am J Dermatopathology. (1993) 15:429–34. doi: 10.1097/00000372-199310000-00003 7694515

[32] Kutzner H . Expression of the human progenitor-cell antigen cd34 (hpca-1) distinguishes dermatofibrosarcoma protuberans from fibrous histiocytoma in formalin-fixed, paraffin-embedded tissue. J Am Acad Dermatol. (1993) 28:613–7. doi: 10.1016/0190-9622(93)70083-6 7681857

[33] Hornick JL Fletcher CDM . Immunohistochemical staining for KIT (CD117) in soft tissue sarcomas is very limited in distribution. Am J Clin Pathol. (2002) 117:188–93. doi: 10.1309/LX9U-F7P0-UWDH-8Y6R 11865845

[34] Mentzel T . Cutaneous mesenchymal tumours: an update. Pathology. (2014) 46:149–59. doi: 10.1097/PAT.0000000000000046 24378387

[35] Schaefer IM Fletcher CDM Hornick JL . Loss of H3K27 trimethylation distinguishes malignant peripheral nerve sheath tumors from histologic mimics. Modern Pathol. (2016) 29:4–13. doi: 10.1038/modpathol.2015.134 26585554

[36] Vivero M Doyle LA Fletcher CDM Mertens F Hornick JL . GRIA2 is a novel diagnostic marker for solitary fibrous tumour identified through gene expression profiling. Histopathology. (2014) 65:71–80. doi: 10.1111/his.2014.65.issue-1 24456377

[37] Hanft VN Shea CR McNutt NS Pullitzer D Horenstein MG Prieto VG . Expression of CD34 in sclerotic ("plywood") fibromas. Am J Dermatopathology. (2000) 22:17–21. doi: 10.1097/00000372-200002000-00003 10698210

[38] Kutzner H Mentzel T Palmedo G Hantschke M Rutten A Paredes BE . Plaque-like CD34-positive Dermal Fibroma ("Medallion-like Dermal Dendrocyte Hamartoma") Clinicopathologic, Immunohistochemical, and Molecular Analysis of 5 Cases Emphasizing its Distinction From Superficial, Plaque-like Dermatofibrosarcoma Protuberans. Am J Surg Pathol. (2010) 34:190–201. doi: 10.1097/PAS.0b013e3181c7cf11 20061935

[39] Rapini RP Golitz LE . Sclerotic fibromas of the skin. J Am Acad Dermatol. (1989) 20:266–71. doi: 10.1016/S0190-9622(89)70033-5 2464630

[40] Salgado R Llombart B M Pujol R Fernández-Serra A Sanmartín O Toll A . Molecular diagnosis of dermatofibrosarcoma protuberans: a comparison between reverse transcriptase-polymerase chain reaction and fluorescence *in situ* hybridization methodologies. Genes Chromosomes Cancer. (2011) 50:510–7. doi: 10.1002/gcc.20874 21484928

[41] Iwasaki T Yamamoto H Oda Y . Current Update on the Molecular Biology of Cutaneous Sarcoma: Dermatofibrosarcoma Protuberans. Curr Treat Options Oncol. (2019) 20:29. doi: 10.1007/s11864-019-0628-3 30874910

[42] Tang X Hu X Wen Y Min L . Progressive insights into fibrosarcoma diagnosis and treatment: leveraging fusion genes for advancements. Front Cell Dev Biol. (2023) 11:1284428. doi: 10.3389/fcell.2023.1284428 37920823 PMC10618559

[43] Zhang X Sun D Zheng H Rao Y Deng Y Liang X . Comprehensive analysis of transcriptome characteristics and identification of TLK2 as a potential biomarker in dermatofibrosarcoma protuberans. Front Genet. (2022) 13. doi: 10.3389/fgene.2022.926282 PMC9483842 36134026

[44] Kambayashi Y Kasahara Y Ohuchi K Amagai R Hashimoto A Asano Y . Successful treatment of metastatic fibrosarcomatous dermatofibrosarcoma protuberans with imatinib mesylate. Dermatol Ther. (2022) 35:e15736. doi: 10.1111/dth.v35.10 35898161

[45] Tatai T Gomi D Fukushima T Kobayashi T Sekiguchi N Sakamoto A . Effectiveness of Imatinib Mesylate Treatment in a Patient with Dermatofibrosarcoma Protuberans with Pulmonary and Pancreatic Metastases. Intern Med. (2016) 55:2507–11. doi: 10.2169/internalmedicine.55.6836 27580559

[46] McArthur GA Demetri GD van Oosterom A Heinrich MC Debiec-Rychter M Corless CL . Molecular and clinical analysis of locally advanced dermatofibrosarcoma protuberans treated with imatinib: Imatinib Target Exploration Consortium Study B2225. J Clin Oncol. (2005) 23:866–73. doi: 10.1200/JCO.2005.07.088 15681532

[47] Kuhlmann C Ehrl D Taha S Wachtel N Schmid A Bronsert P . Dermatofibrosarcoma protuberans of the scalp: Surgical management in a multicentric series of 11 cases and systematic review of the literature. Surgery. (2023) 173:1463–75. doi: 10.1016/j.surg.2023.02.026 37012145

[48] Xiong JX Cai T Hu L Chen XL Huang K Chen AJ . Risk factors related to postoperative recurrence of dermatofibrosarcoma protuberans: A retrospective study and literature review. World J Clin cases. (2021) 9:5442–52. doi: 10.12998/wjcc.v9.i20.5442 PMC8281415 34307598

[49] Lowe GC Onajin O Baum CL Otley CC Arpey CJ Roenigk RK . A Comparison of Mohs Micrographic Surgery and Wide Local Excision for Treatment of Dermatofibrosarcoma Protuberans With Long-Term Follow-up: The Mayo Clinic Experience. Dermatologic Surg. (2017) 43:98–106. doi: 10.1097/DSS.0000000000000910 27749444

[50] Malan M Xuejingzi W Quan SJ . The efficacy of Mohs micrographic surgery over the traditional wide local excision surgery in the cure of dermatofibrosarcoma protuberans. Pan Afr Med J. (2019) 33. doi: 10.11604/pamj.2019.33.297.17692 PMC6815477 31692830

[51] Chen J Sun D Rao Y-M Zheng H-Y Gong X Xu H . Practice for multidisciplinary diagnosis and treatment of dermatofibrosarcoma protuberans: expert consensus of Shanghai Ninth People's Hospital, Shanghai Jiao Tong University School of Medicine, (2020 edition). J Of Shanghai Jiao Tong Univ. (2021) 41:1669–75.

[52] Behan FC Rozen WM Kwee MM Kapila S Fairbank S Findlay MW . Oncologic clearance with preservation of reconstructive options: literature review and the 'delayed reconstruction after pathology evaluation (DRAPE)' technique. ANZ J Surg. (2012) 82:780–5. doi: 10.1111/j.1445-2197.2012.06265.x 22984967

[53] Vandeweyer E Seyeidi JV Deraemaecker R . Dermatofibrosarcoma protuberans of the upper lip: An overview and a case report. Eur J Surg Oncol. (1997) 23:275–7. doi: 10.1016/S0748-7983(97)92700-1 9236907

[54] Bowne WB Antonescu CR Leung DH Katz SC Hawkins WG Woodruff JM . Dermatofibrosarcoma protuberans: A clinicopathologic analysis of patients treated and followed at a single institution. Cancer. (2000) 88:2711–20. doi: 10.1002/1097-0142(20000615)88:12<2711::AID-CNCR9>3.0.CO;2-M 10870053

[55] Liang CA Jambusaria-Pahlajani A Karia PS Elenitsas R Zhang PD Schmults CD . A systematic review of outcome data for dermatofibrosarcoma protuberans with and without fibrosarcomatous change. J Am Acad Dermatol. (2014) 71:781–6. doi: 10.1016/j.jaad.2014.03.018 24755121

[56] Heymann WR . Dermatofibrosarcoma protuberans recurrence: Size matters. J Am Acad Dermatol. (2023) 89:909–10. doi: 10.1016/j.jaad.2023.09.001 37678497

[57] Marcoval J Moreno-Vílchez C Torrecilla-Vall-Llossera C Muntaner-Virgili C Pérez Sidelnikova D Sanjuán X . Dermatofibrosarcoma protuberans. A study of 148 patients. Dermatology. (2024) 240:487–493. doi: 10.1159/000536172 PMC11168446 38228098

[58] Ishizuki S Nakamura Y . Evidence from Clinical Studies Related to Dermatologic Surgeries for Skin Cancer. Cancers. (2022) 14:27. doi: 10.3390/cancers14153835 PMC9367341 35954498

[59] Llombart B Serra C Requena C Alsina M Morgado-Carrasco D Través V . Guidelines for Diagnosis and Treatment of Cutaneous Sarcomas: Dermatofibrosarcoma Protuberans. Actas Dermosifiliogr (Engl Ed). (2018) 109:868–77. doi: 10.1016/j.ad.2018.05.006 30539729

[60] Chen Y Shi YZ Feng XH Wang XT He XL Zhao M . Novel TNC-PDGFD fusion in fibrosarcomatous dermatofibrosarcoma protuberans: a case report. Diagn Pathol. (2021) 16:63. doi: 10.1186/s13000-021-01123-1 34256767 PMC8276425

[61] Schmults CD Blitzblau R Aasi SZ Alam M Alam M Baumann BC . NCCN Clinical Practice Guidelines in Oncology-Dermatofibrosarcoma Protuberans National Comprehensive Cancer Network® (2022). Available online at: http://www.nccn.org/. 10.6004/jnccn.2025.0001 39819674

[62] McKee PH Fletcher CD . Dermatofibrosarcoma protuberans presenting in infancy and childhood. J Cutan Pathol (1991) 18(4):241–6 10.1111/j.1600-0560.1991.tb01230.x 1939782

[63] Han QQ Chen M Yang JL Du TH Peng H . Dermatofibrosarcoma protuberans of the face: A clinicopathologic and molecular study of 34 cases. J Dtsch Dermatol Ges (1922) 20(11):1463–73 10.1111/ddg.14882 36377270

[64] Tillman BN Liu JC . Cutaneous Sarcomas. Otolaryngol Clin N Am (2021) 54(2):369–78 10.1016/j.otc.2020.11.010 33602520

[65] Baig IT Lauck K Nguyen Q-BD . Tumor size is the most significant risk factor for local recurrence in dermatofibrosarcoma protuberans: A large-scale retrospective cohort analysis. J Am Acad Dermatol (2023) 89(5):1054–4 10.1016/j.jaad.2023.06.044 37419185

[66] Bogucki B Neuhaus I Hurst EA . Dermatofibrosarcoma Protuberans: A Review of the Literature. Dermatol Surg (2012) 38(4):537–51 10.1111/j.1524-4725.2011.02292.x 22288484

[67] Lim SX Ramaiya A Levell NJ Venables ZC . Review of dermatofibrosarcoma protuberans. Clin Exp Dermatol (2023) 48(4):297–302 10.1093/ced/llac111 36630365

[68] Ding X Yang Z . Knowledge mapping of platform research: a visual analysis using VOSviewer and CiteSpace. Electronic Commerce Research (2020) 22(3):787–809

[69] Merigó JM Yang J-B . A bibliometric analysis of operations research and management science. Omega (2017) 73:37–48

[70] Patel KU Szabo SS Hernandez VS Prieto VG Abruzzo LV Lazar AJF . Dermatofibrosarcoma protuberans COL1A1-PDGFB fusion is identified in virtually all dermatofibrosarcoma protuberans cases when investigated by newly developed multiplex reverse transcription polymerase chain reaction and fluorescence in situ hybridization assays. Hum Pathol (2008) 39(2):184–93 10.1016/j.humpath.2007.06.009 17950782

[71] Llombart B Serra-Guillen C Monteagudo C Guerrero JAL Sanmartin O . Dermatofibrosarcoma protuberans: a comprehensive review and update on diagnosis and management. Semin Diagn Pathol (2013) 30(1):13–28 10.1053/j.semdp.2012.01.002 23327727

[72] Maki RG Awan RA Dixon RH Jhanwar S Antonescu CR . Differential sensitivity to imatinib of 2 patients with metastatic sarcoma arising from dermatofibrosarcoma protuberans. Int J Cancer (2002) 100(6):623–6 10.1002/ijc.10535 12209598

[73] Stacchiotti S Pedeutour F Negri T Conca E Marrari A Palassini E . Dermatofibrosarcoma protuberans-derived fibrosarcoma: clinical history, biological profile and sensitivity to imatinib. Int J Cancer (2011) 129(7):1761–72 10.1002/ijc.25826 21128251

[74] Dickson BC Hornick JL Fletcher CDM Demicco EG Howarth DJ Swanson D . Dermatofibrosarcoma protuberans with a novel COL6A3-PDGFD fusion gene and apparent predilection for breast. Gene Chromosomes Cancer (2018) 57(9):437–45 10.1002/gcc.22663 PMC6762016 30014607

[75] Maloney N Bridge JA De abreu F Korkolopoulou P Sakellariou S Linos K . A novel MAP3K7CL-ERG fusion in a molecularly confirmed case of dermatofibrosarcoma protuberans with fibrosarcomatous transformation. J Cutan Pathol (2019) 46(7):532–7 10.1111/cup.13469 30950098

[76] Peng C Jian XX Xie Y Li LF Ouyang J Tang L . Genomic alterations of dermatofibrosarcoma protuberans revealed by whole-genome sequencing. Br J Dermatol (2022) 186(6):997–1009 10.1111/bjd.20976 PMC9325047 35441365

[77] Kosela-paterczyk H Rutkowski P . Dermatofibrosarcoma protuberans and gastrointestinal stromal tumor as models for targeted therapy in soft tissue sarcomas. Expert Rev Anticancer Ther (2017) 17(12):1107–16 10.1080/14737140.2017.1390431 28988501

[78] Noujaim J Thway K Fisher C Jones RL . Dermatofibrosarcoma protuberans: from translocation to targeted therapy. Cancer Biol Med (2015) 12(4):375–84 10.7497/j.issn.2095-3941.2015.0067 PMC4706526 26779374

[79] Sugiura H Fujiwara Y Ando M Kawai A Ogose A Ozaki T . Multicenter Phase II trial assessing effectiveness of imatinib mesylate on relapsed or refractory KIT-positive or PDGFR-positive sarcoma. J Orthop Sci (2010) 15(5):654–60 10.1007/s00776-010-1506-9 20953927

[80] Rubin BP Schuetze SM Eary JF Norwood TH Mirza S Conrad EU . Molecular targeting of platelet-derived growth factor B by imatinib mesylate in a patient with metastatic dermatofibrosarcoma protuberans. J Clin Oncol (2002) 20(17):6586–91 10.1200/JCO.2002.01.027 12202658

[81] Hiraki-hotokebuchi Y Yamada Y Kohashi K Yamamoto H Endo M Setsu N . Alteration of PDGFR beta-Akt-mTOR pathway signaling in fibrosarcomatous transformation of dermatofibrosarcoma protuberans. Hum Pathol (2017) 67:60–8 10.1016/j.humpath.2017.07.001 28711648

[82] Ugurel S Mentzel T Utikal J Helmbold P Mohr P Pfohler C . Neoadjuvant Imatinib in Advanced Primary or Locally Recurrent Dermatofibrosarcoma Protuberans: A Multicenter Phase II DeCOG Trial with Long-term Follow-up. Clin Cancer Res (2014) 20(2):499–510 10.1158/1078-0432.CCR-13-1411 24173542

[83] Eilers G Czaplinski JT Mayeda M Bahri N Tao D Zhu MJ . CDKN2A/p16 Loss Implicates CDK4 as a Therapeutic Target in Imatinib-Resistant Dermatofibrosarcoma Protuberans. Mol Cancer Ther (2015) 14(6):1346–53 10.1158/1535-7163.MCT-14-0793 PMC4458458 25852058

[84] Smola MG Soyer HP Scharnagl E . Surgical-treatment of dermatofibrosarcoma protuberans - a retrospective study of 20 cases with review of literature. Eur J Surg Oncol (1991) 17(5):447–53 1936291

[85] Hafner HM Moehrle M Eder S Trilling B Rocken M Breuninger H . 3D-Histological evaluation of surgery in dermatofibrosarcoma protuberans and malignant fibrous histiocytoma: Differences in growth patterns and outcome. Ejso (2008) 34(6):680–6 10.1016/j.ejso.2007.07.004 17716851

[86] Charalambides M Yannoulias B Malik N Mann JK Celebi P Veitch D . A review of Mohs micrographic surgery for skin cancer. Part 1: Melanoma and rare skin cancers. Clin Exp Dermatol (2022) 47(5):833–49 10.1111/ced.15081 34939669

[87] Veronese F Boggio P Tiberio R Gattoni M Fava P Caliendo V . Wide local excision vs. Mohs Tubingen technique in the treatment of dermatofibrosarcoma protuberans: a two-centre retrospective study and literature review. J Eur Acad Dermatol Venereol (2017) 31(12):2069–76 10.1111/jdv.14378 28573714

[88] Dubay D Cimmino V Lowe L Johnson TM Sondak VK . Low recurrence rate after surgery for dermatofibrosarcoma protuberans - A multidisciplinary approach from a single institution. Cancer (2004) 100(5):1008–16 10.1002/cncr.20051 14983497

[89] Durack A Gran S Gardiner MD Jain A Craythorne E Proby CM . A 10-year review of surgical management of dermatofibrosarcoma protuberans. Br J Dermatol (2021) 184(4):731–9 10.1111/bjd.19346 32599647

[90] Foroozan M Sei JF Amini M Beauchet A Saiag P . Efficacy of Mohs Micrographic Surgery for the Treatment of Dermatofibrosarcoma Protuberans Systematic Review. Arch Dermatol (2012) 148(9):1055–63 10.1001/archdermatol.2012.1440 22986859

[91] Gloster HM Harris KR Roenigk RK . A comparison between Mohs micrographic surgery and wide surgical excision for the treatment of dermatofibrosarcoma protuberans. J Am Acad Dermatol (1996) 35(1):82–7 8682970

[92] Meguerditchian AN Wang JP Lema B Kraybill WG Zeitouni NC Kane JM . Wide Excision or Mohs Micrographic Surgery for the Treatment of Primary Dermatofibrosarcoma Protuberans. Am J Clin Oncol-Cancer Clin Trials (2010) 33(3):300–3 10.1097/COC.0b013e3181aaca87 19858696

[93] Paradisi A Abeni D Rusciani A Cigna E Wolter M Scuderi N . Dermatofibrosarcoma protuberans: Wide local excision vs. Mohs micrographic surgery. Cancer Treat Rev (2008) 34(8):728–36. 10.1016/j.ctrv.2008.06.002 18684568

[94] Garcia C Viehman G Hitchcock M Clark RE . Dermatofibrosarcoma protuberans treated with mohs surgery - A case with CD34 immunostaining variability. Dermatol Surg (1996) 22(2):177–9. 10.1111/j.1524-4725.1996.tb00503.x 8608381

[95] Lemm D Mugge LO Mentzel T Hoffen K . Current treatment options in dermatofibrosarcoma protuberans. J Cancer Res Clin Oncol (2009) 135(5):653–65. 10.1007/s00432-009-0550-3 PMC12160171 19205737

[96] Massey RA Tok J Strippoli BA Szabolcs MJ Silvers DN Eliezri YD . Current treatment options in dermatofibrosarcoma protuberans. J Cancer Res Clin Oncol (2009) 135(5):653–65. 10.1007/s00432-009-0550-3 PMC12160171 19205737

[97] Zongo N Guigemde RA Yameogo PB Some RO Traore B Dem A . Dermatofibrosarcoma protuberans surgery: Experiences of four African surgical oncology units and literature review. J Surg Oncol (2022) 1226(8):1512–9. 10.1002/jso.27077 35997990

[98] Cui L Wang GF Li X Song YQ Pu WW Zhang D . Modified low-dose second window indocyanine green technique improves near-infrared fluorescence image-guided dermatofibrosarcoma protuberans resection: A randomized control trial. Front Surg (2022) 9:11. 10.3389/fsurg.2022.984857 PMC9684718 36439528

[99] Abbott JJ Oliveira AM Nascimento AG . The prognostic significance of fibrosarcomatous transformation in dermatofibrosarcoma protuberans. Am J Surg Pathol (2006) 30(4):436–43. 10.1097/00000478-200604000-00002 16625088

[100] Hoesly PM Lowe GC Lohse CM Brewer JD Lehman JS . Prognostic impact of fibrosarcomatous transformation in dermatofibrosarcoma protuberans: A cohort study. J Am Acad Dermatol (2015) 72(3):419–25. 10.1016/j.jaad.2014.11.020 25582537

[101] Llombart B Monteagudo C Sanmartin O Lopez-Guerrero JA Serra-Guillen C Poveda A . Dermatofibrosarcoma protuberans: A clinicopathological, immunohistochemical, genetic (COL1A1-PDGFB), and therapeutic study of low-grade versus high-grade (fibrosarcomatous) tumors. J Am Acad Dermatol (2011) 65(3):564–75. 10.1016/j.jaad.2010.06.020 21570152

[102] Mallett KE Almubarak S Claxton RM Ferguson PC Griffin AM Rose PS . Preoperative Risk Factors for Fibrosarcomatous Transformation in Dermatofibrosarcoma Protuberans. Anticancer Res (2022) 42(1):105–8. 10.21873/anticanres.15463 34969715

[103] Li YN Wang C Xiang B Chen SY Li L Ji Y . Clinical Features, Pathological Findings and Treatment of Recurrent Dermatofibrosarcoma Protuberans. J Cancer (2017) 8(7):1319–23. 10.7150/jca.17988 PMC5463448 28607608

[104] Mentzel T Beham A Katenkamp D Tos APD Fletcher CDM . Fibrosarcomatous ("high-grade") dermatofibrosarcoma protuberans - Clinicopathologic and immunohistochemical study of a series of 41 cases with emphasis on prognostic significance. Am J Surg Pathol (1998) 22(5):576–87. 10.1097/00000478-199805000-00009 9591728

[105] Dadone-Montaudie B Alberti L Duc A Delespaul L Lesluyes T Perot G . Alternative PDGFD rearrangements in dermatofibrosarcomas protuberans without PDGFB fusions. Mod Pathol (2018) 31(11):1683–93. 10.1038/s41379-018-0089-4 29955147

[106] Lu B Li L Chen A Peng H Du TH Qiu Y . Coamplification of 12q15 and 12p13 and homozygous CDKN2A/2B deletion: synergistic role of fibrosarcomatous transformation in dermatofibrosarcoma protuberans with a cryptic COL1A1-PDGFB fusion. Virchows Arch (2022) 481(2):313–9. 10.1007/s00428-022-03297-5 35171326

[107] Augsburger D Nelson J Kalinski T Udelnow A Knosel T Hofstetter M . Current diagnostics and treatment of fibrosarcoma -perspectives for future therapeutic targets and strategies. Oncotarget (2017) 8(61):104638–53. 10.18632/oncotarget.20136 PMC5732833 29262667

