# Supplementary figures and images for: Hotspots and future trends of dermatofibrosarcoma protuberans
Source: Front Oncol. 2024 Nov 12;14:1399486. doi: 10.3389/fonc.2024.1399486 (PMC11588712; doi:10.3389/fonc.2024.1399486)

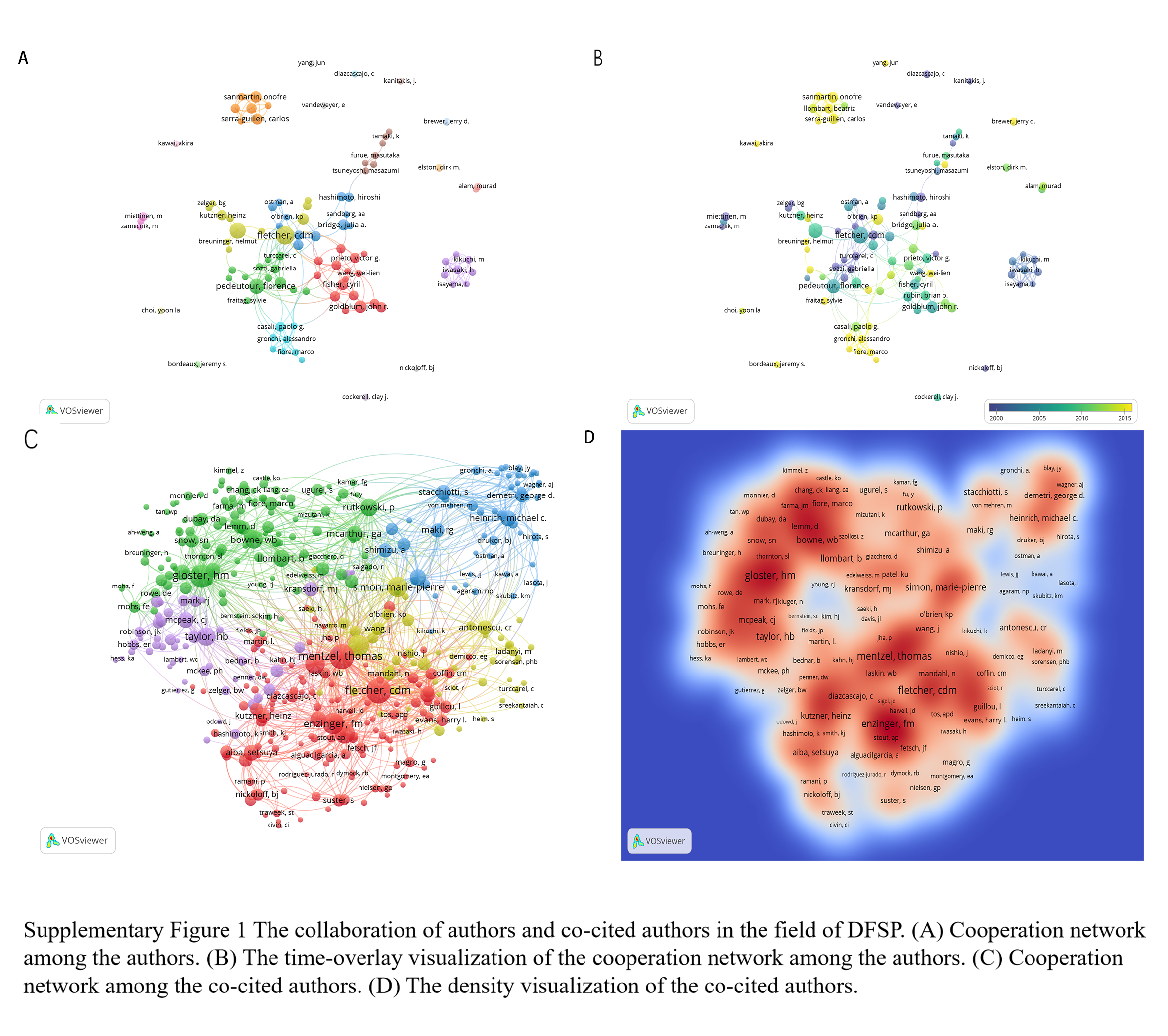

Supplement: Supplementary file 1 [file Image1.tif]

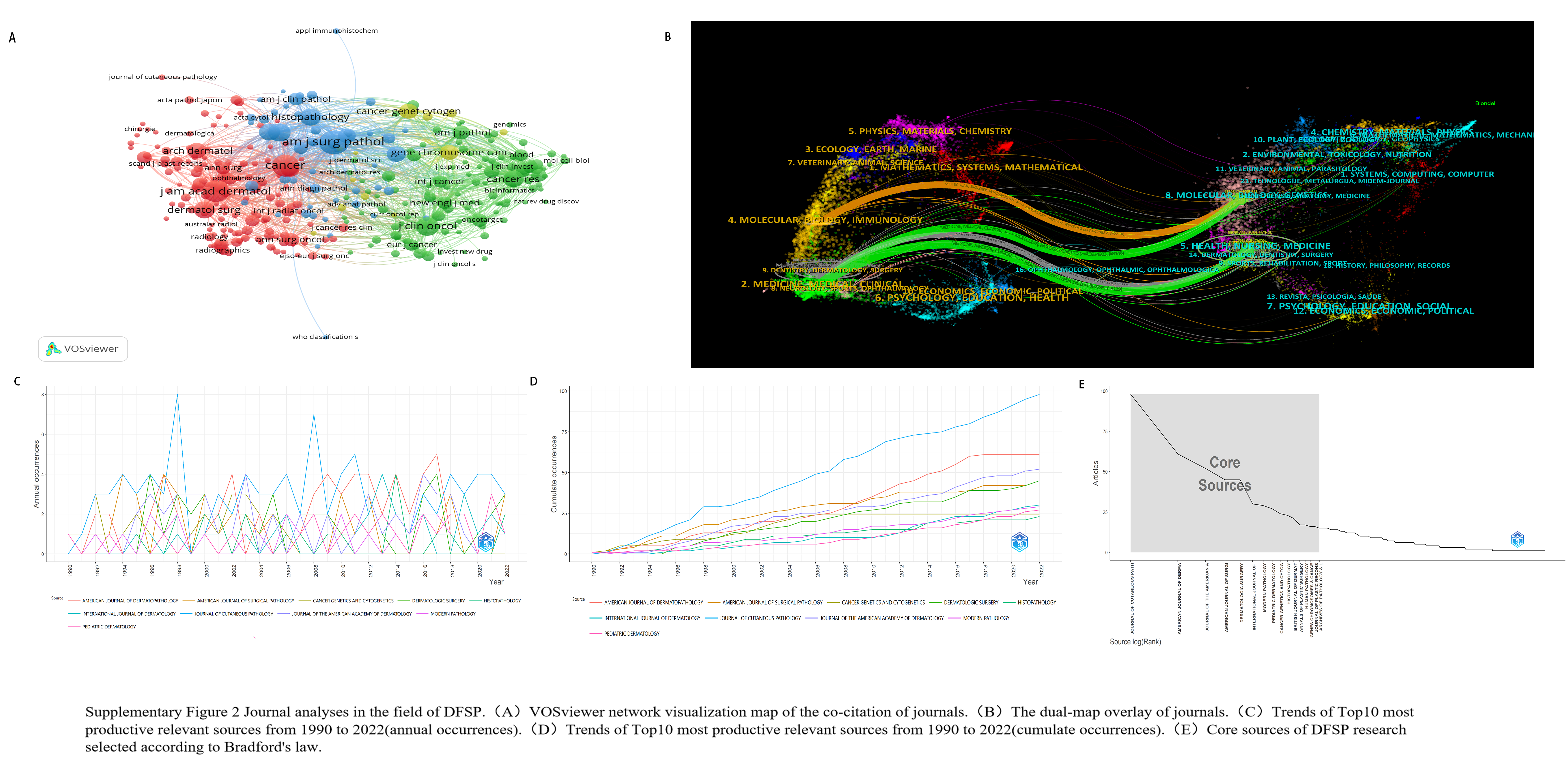

Supplement: Supplementary file 2 [file Image2.tif]

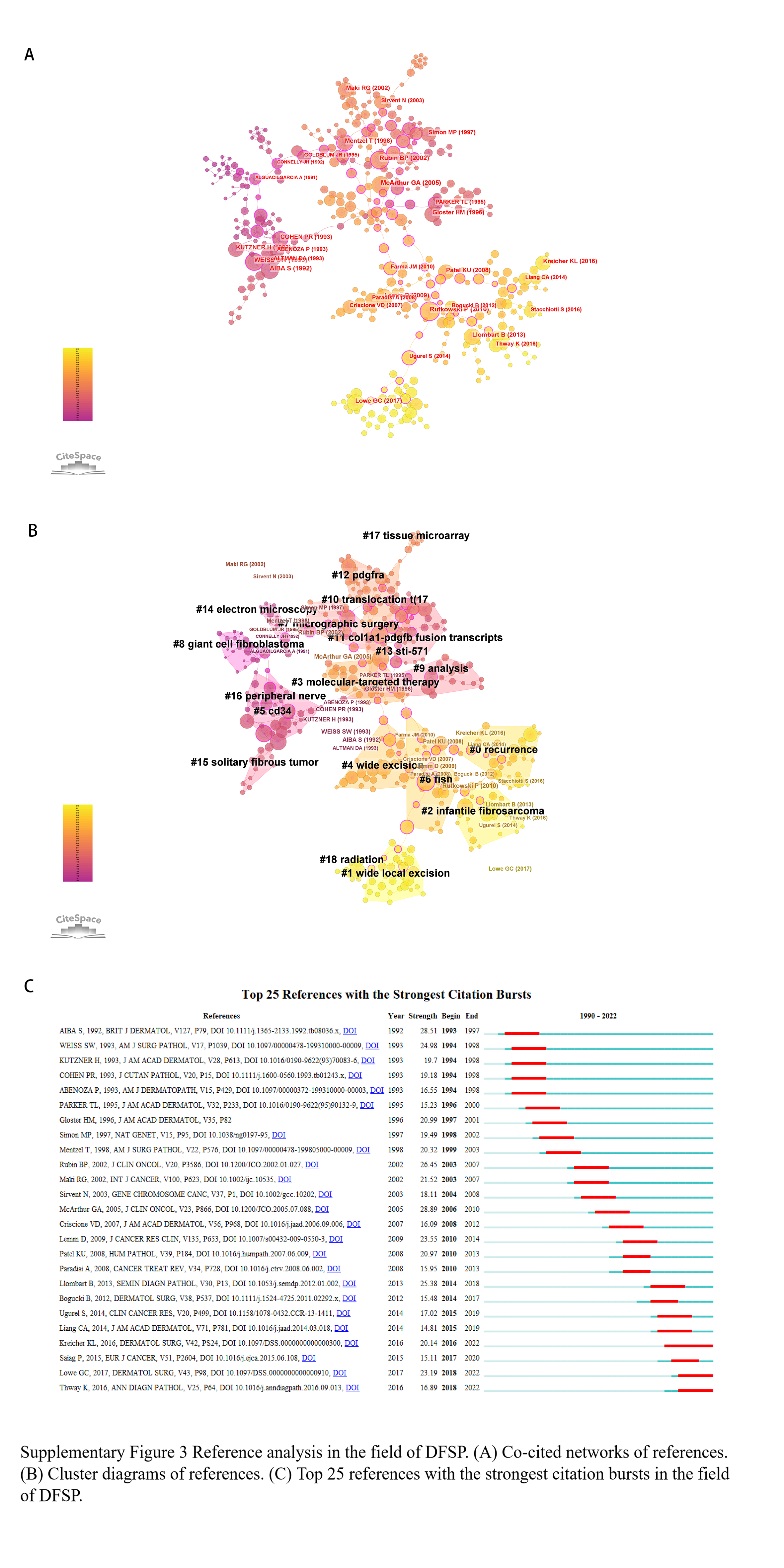

Supplement: Supplementary file 3 [file Image3.tif]
